# Wearables for Industrial Work Safety: A Survey

**DOI:** 10.3390/s21113844

**Published:** 2021-06-02

**Authors:** Ekaterina Svertoka, Salwa Saafi, Alexandru Rusu-Casandra, Radim Burget, Ion Marghescu, Jiri Hosek, Aleksandr Ometov

**Affiliations:** 1 Department of Telecommunications, Faculty of Electronics, Telecommunications and Information Technology, University Politehnica of Bucharest, 061071 Bucharest, Romania; alexandru.rusu@upb.ro (A.R.-C.); ion.marghescu@upb.ro (I.M.); 2 Department of Telecommunications, Faculty of Electrical Engineering and Communication, Brno University of Technology, Technicka 12, 616 00 Brno, Czech Republic; saafi@feec.vutbr.cz (S.S.); burgetrm@feec.vutbr.cz (R.B.); hosek@feec.vutbr.cz (J.H.); 3 Unit of Electrical Engineering, Faculty of Information Technology and Communication Sciences, Tampere University, 33720 Tampere, Finland; aleksandr.ometov@tuni.fi

**Keywords:** wearables, smart devices, occupational safety, IIoT, data collection, communications, localization

## Abstract

Today, ensuring work safety is considered to be one of the top priorities for various industries. Workplace injuries, illnesses, and deaths often entail substantial production and financial losses, governmental checks, series of dismissals, and loss of reputation. Wearable devices are one of the technologies that flourished with the fourth industrial revolution or Industry 4.0, allowing employers to monitor and maintain safety at workplaces. The purpose of this article is to systematize knowledge in the field of industrial wearables’ safety to assess the relevance of their use in enterprises as the technology maintaining occupational safety, to correlate the benefits and costs of their implementation, and, by identifying research gaps, to outline promising directions for future work in this area. We categorize industrial wearable functions into four classes (monitoring, supporting, training, and tracking) and provide a classification of the metrics collected by wearables to better understand the potential role of wearable technology in preserving workplace safety. Furthermore, we discuss key communication technologies and localization techniques utilized in wearable-based work safety solutions. Finally, we analyze the main challenges that need to be addressed to further enable and support the use of wearable devices for industrial work safety.

## 1. Introduction

The workplace is fraught with many sources of danger, especially in enterprises with harmful work conditions. For a long time, the work safety issue has been relegated to the background by employers for the sake of labor productivity. Published by World Health Organization (WHO) [1], statistics on industrial death accidents from 1970 to the present day have a shape close to Gaussian. The lack of statistics can explain this observation since only six countries maintained such a base starting from the 70th. However, the emergence of new technologies, including wearable devices, can also contribute to constraining mortality in industries nowadays [2].

Although the number of accidents per year tends to decrease, the level of mortality in workplaces is still considerable. According to the International Labor Organization (ILO) [3], approximately 1.9 million people have work-related diseases, and 2.3 million people die from work accidents annually. Besides, these statistics reflect only reported cases: not all enterprises openly register all cases, thus, not entailing inspections, sanctions, unrest among staff, loss of reputation, etc. Therefore, at least 4.2 million people suffer in the workplace per year, and 45% of countries have a population less than this number [4].

The problem of work safety in industrial environments is still on the crest of a wave. Worldwide statistics show a high rate of death and injury at work, a variety of hazardous industries, and sources of danger [3]. With the advent of Industry 4.0 and broad integration of the Internet of Things (IoT), employers are expected to achieve better safety mainly due to the emergence of various technologies [5]. This paper is primarily focusing on the smallest form-factor personal devices, namely, wearables, that also attempt to achieve the same goal as part of the Internet of Wearable Things (IoWT) paradigm [6]. Further discussion will focus on the Industrial IoT (IIoT) that emerged to design, maintain, monitor, optimize, and analyze industrial operations to gain real-time insights, make effective decisions and maintain occupational safety [7,8].

Historically, the IIoT was at the initial stage of its development as of 2015 [9]. At this point, many entrepreneurs had doubts about the feasibility of introducing such an innovation due to the uncertainty about the impact that it will have on workers, labor processes, production, and, more importantly, profits. The situation has begun to change in the last five years. According to [7], the IIoT market size is estimated at 77 billion USD, with a perspective reaching 110 billion USD by 2025. However, this forecast was delivered before the spread of the COVID-19. Due to the global pandemic situation, many enterprises terminated their businesses or even claimed bankruptcy. Indirectly, we can estimate a decrease in production capacity by an increase in unemployment. For example, one of the most significant blows hit the manufacturing, construction, transportation, and storage sectors according to the UK statistics [10,11]. In contrast, some industrial wearable device manufacturers took advantage of the situation by adjusting products to the circumstances. For example, Estimote has redesigned its industrial wearable tracking devices to remember contacts between workers closer than two meters [11,12].

By meeting two basic requirements for any IoT device, namely, access to the Internet and communication solutions, wearables have become one of the most important IoT concepts, forming IoWT as a promising yet young segment. Various forecasts state that the wearable device market will reach 57 billion USD by 2022 [13], or even 64 billion USD by 2025 [14], and 104 billion USD by 2027 [15]. Wristbands and bracelets currently occupy the leading position among wearable devices and smartwatches, which market share is almost 50% [16].

As of today, research literature still lacks comprehensive reviews on wearable technology and its industrial utilization [17]. The most solid work considering this topic is [18]. In this paper, the authors distinguished 24 categories of wearable technologies and divided them into five groups depending on the functions; monitoring, tracking, augmenting, assisting, and delivering content. Moreover, they highlight six motivations behind the use of wearable devices in industrial environments: the ability to monitor employees’ psychological and physiological factors, enhance operational efficiency, promote work environment safety and security, and improve workers’ health. Finally, they revealed the main challenge groups compliant with the adoption of wearable devices; technological challenges (trade-off between size, weight, battery functions, accuracy, etc.), social challenges (confidentiality of data, lack of technical skills, high dependency on the wearable device), policies and standards set by governments, economic challenges (high cost of the wearable devices and its integration with other systems), and data challenges (data ownership issue, huge amount of data).

**Figure 1:**
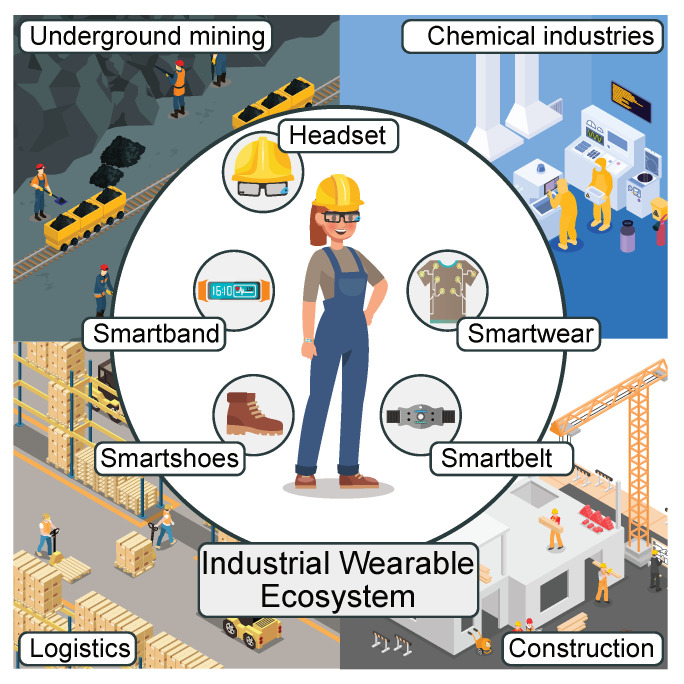
The concept of using wearable devices in industries to maintain work safety.

In particular, authors in [19] review wearable devices as part of the IoT concept, mentioning work safety in the list of areas where this technology is beneficial but without special focus on it. On the opposite, some other works explore the use of wearable devices in a narrow specific area of the industry. Moreover, the authors of [20] consider the mining industry, while work [21] deals with the construction industry. However, to the best of the author’s knowledge, none of these works investigates industrial wearables focusing on occupational safety or reviews key aspects of data collection, data transmission, and localization. Driven by the works mentioned above, this paper aims to analyze and integrate information related to wearable devices and provides a comprehensive overview of the different features of their use in maintaining and increasing work safety in potentially hazardous industries, as depicted in Figure 1.

For this work, we formulated the following research questions:


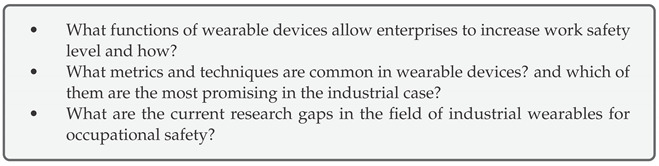


To find the answers to our research questions, a widely-used methodology was adopted to carry out a systematic literature review based on the PRISMA [22]. To identify key publications on the analysis of wearable technology for work safety, we performed a literature search in scientific databases that cover leading computer science journals and conferences including *IEEE Xplore*, *ACM Digital Library*, *ScienceDirect*, *SAGE Journals Online*, and *Springer Link*. To find relevant articles and papers for our research, we applied the following search string: (Wearable OR “Body Area”) AND (Safety OR Industr* OR Injury) for the past five years and in the fields of Electrical Engineering, Applied Physics, Telecommunications, Biomedical Engineering, and Computer Information Systems. In total, we gathered a set of 1290 potentially relevant publications, excluding grey literature and pre-prints.

We then analyzed the titles, keywords, and abstracts of the publications to identify papers and articles that describe at least one modeling or simulation approach for distributed ledgers. While doing so, we selected a total of 75 publications. To further extend our literature sample, we analyzed the selected publications’ references for additional papers relevant to our research. Following this process, it resulted in a total of 68 publications. The literature list was further extended based on the additional references identified in the bodies of the selected ones or referencing those. Once the literature selection process was completed, we carefully read the selected publications to identify the described applications and challenges. The results of our analysis form the core of the topical literature review and are presented in the following sections.

The rest of this article is organized as follows. Section 2 discusses industrial wearables in more detail, considering main groups, functions, examples of applications, and benefits to the industry in general and from an occupational safety perspective in particular. Next, Section 3 highlights metrics, technologies, and approaches regarding the techniques of data collection, data transmission, and localization. Further, Section 4 provides the main challenges and future perspectives of this work. The last section concludes the paper.

## 2. Industrial Wearable Devices

The IIoT provides a wider view and a deeper understanding of the company’s processes by integrating different sensors, wearables, software, and data processing tools. The clearest advantage of wearables in IIoT is lucre, which is reached by increasing operational efficiency, reducing downtime, and optimizing business processes. Less frequently discussed in the literature, the benefit is related to how wearable technology can maintain workplace safety. It is necessary to identify the main sources of security threats and the causes of workplace accidents to answer the first research question. This paper, first of all, identifies the most dangerous industrial sectors in the world.

**Figure 2:**
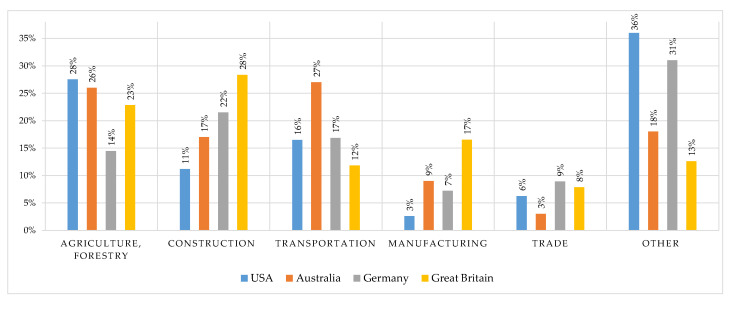
The rate of deaths due to work-related accidents during 2014–2020 (for approx. 100,000).

There are no general statistics on mortality from injuries by the industry sector. However, some countries keep such records providing statistics in ratios (commonly, the number of deaths per 100,000 workers) without mentioning the actual number of accidents. Hopefully, in the near future, the management of statistics in enterprises will be more widespread and, importantly, standardized so that data from different places can be easily compared, problems—identified, and experience in dealing with them—shared. Figure 2 outlines the results of investigations conducted in USA [23], Australia [24], Germany [25], Great Britain [26].

The most dangerous identified industry sectors are agriculture/forestry, construction, transportation, manufacturing, and trade. Due to differences in classification, most sectors such as mining, oil and gas extraction, and recycling fall under the “other” category. Notably, the four key causes in decreasing order of frequency are: fall from height, struck by moving/falling object, caught-in/between (when a worker is between the parts of machinery/object [27]), and hit by moving vehicle. Among the other reasons are also cuts, car crashes, reaction to improper motion, electrocutions, hazardous substances, chemicals [20,28]. According to [1], the main consequences that lead to death due to the last two reasons are chronic obstructive pulmonary disease and cancer (more often, lung cancer and mesothelioma). Nonetheless, constant stress should also be added to the list. Over time, employers have become more concerned with maintaining a favorable working atmosphere, but the impact of stress on human health is still underestimated in many organizations. In the short term, it may lead to various disorders, from chronic fatigue to depression. In the long-term, it may entail severe psychological problems and several problems such as the higher risk of myocardial ischemia, cardiac arrhythmia, anorexia, Alzheimer’s disease, insomnia, etc. [29,30].

Importantly, industrial wearables have increased requirements for reliability. Harsh industrial surroundings, characterized by extreme environmental values (extra-low/high temperatures, high radiation level, etc.), require the wearable device’s physical durability. Also, specific worksites impose the need to develop and improve the accuracy, range, response time, and robustness of traditional technologies. Wearables are one of the most promising solutions to eliminate or reduce the risk of both physical and mental accidents in workplaces. Currently, wearables are widely applied in enterprises and perform many tasks that improve the level of safety at work. Table 1 provides an answer to the first research question by identifying wearable features and functions that help maintain occupational safety.

All industrial wearables functions can be categorized into four groups: monitoring, supporting, training, and tracking. The informing function providing just-in-time information at the workplace and proposed in [18] is seldom represented as a standalone function nowadays and can be, thus, merged with the supporting function. Table 2 gives examples of wearable solutions currently applied in the most hazardous industry branches.

In summary, industries are replete with hazard sources resulting in a high work mortality rate. However, work safety levels can be increased by using wearable devices through the ten functions mentioned above.

## 3. State-of-the-Art Techniques in the Field of Industrial Wearables

The use of a wearable device in the functions discussed above involves other technical aspects, such as data collection, data transfer technologies, and localization methods. This section reviews the existing approaches, pointing out the most promising ones for industrial uses.

### 3.1. Data Collection and Wearable Metrics

The monitoring function is based on the collection of several metrics. In fact, no classification of metrics collected by wearable devices is currently available in research works. We, thus, divide these metrics into two groups depending on the data collected from wearables. The first group is related to the data “extracted” from the human body, and the second group deals with the environment’s information. Table 3 and Table 4 represent the most common wearable metrics and their classification.

In industrial scenarios and setups, the most important and frequent metrics related to the human body are temperature, heart rate, and location. Less commonly used metrics are motion and perspiration. However, the motion metric is essential for industries associated with lifting heavy loads (construction, logistics), and the perspiration can also be relevant for industries with a high probability of heat stress (e.g., mining) [44]. To the best of the authors’ knowledge, the rest of body-related metrics such as EEG, EMG, ECG, heart sounds, sleeping activity, etc. are not tracked in the industries yet, but they can help estimate both the worker’s physical and mental readiness for the industrial process. Developing a comprehensive, lightweight, wearable solution consisting of multiple sensors capable of measuring the human body’s vital parameters as possible will make a significant contribution to eliminating accidents due to human error in hazardous industries. Regarding the parameters related to the environment, it is hard to say what metrics are more frequently used. The industrial conditions define wearable devices’ choice and, consequently, the metrics that can be collected. In the reviewed literature, temperature, relative humidity, and air quality are often used as environment metrics.

### 3.2. Data Transmission

Historically, wearable solutions that appeared in the medical domain were based on a wired communication architecture, where wearable devices transmit their collected data to external processing units via wired links [44,118]. However, relying on wired connectivity restricts user mobility. This limitation was the main reason behind considering the wireless alternative for wearable communications. The migration from wired to wireless connectivity for data transmission is a trend in healthcare monitoring systems and industrial wearables in general. On top of the industrial wearable applications that are provided in Table 2, several examples were studied in the literature and utilized different wireless communication technologies [119,120,121]. These technologies can be classified based on various metrics, among which we choose the range. As a result, we provide in Table 5 a summary of the main short-range, mid-range, and long-range connectivity solutions currently employed in industrial wearable systems.

Due to the battery lifetime consideration, most market-available wearable devices generally rely on smartphone-aided operations using short-range and mid-range communication technologies. In this architecture, the smartphone pre-processes the data sent by the wearable device and acts as a gateway to transmit the pre-processed data to the cloud (if needed). The short-range and mid-range connectivity solutions in industrial wearable applications include Radio Frequency Identification (RFID), ZigBee, Bluetooth, Bluetooth Low Energy (BLE), and Wireless Fidelity (Wi-Fi) [122]. For instance, Reactec company has designed a wearable wristband that measures the amount of Hand-Arm Vibration (HAV) that provides real-time monitoring and automated reporting of HAV exposure [123]. This solution is called HAVWEAR and is utilized by several construction companies to prevent HAV-related diseases such as the white finger syndrome and, thus, to protect the workers’ health conditions. Each wristband contains an RFID card indicating the personalized exposure threshold that each operator should respect [123].

ZigBee and Bluetooth are among the Industrial Wireless Sensor Networks (IWSN) technologies based on the IEEE 802.15 standard and are characterized by the low energy consumption and the support of several topologies [124]. In [125], the authors proposed a wearable system that utilizes ZigBee technology and aims at improving the worker’s safety in the energy industry. As depicted in Table 5, examples of Wi-Fi medium-range standards include IEEE 802.11b, IEEE 802.11g, IEEE 802.11n, and IEEE 802.11ac.

Several life insurance companies offer Intel Basis Peak smartwatches to their customers to measure their heart rates, sleep patterns, and physical activities [123]. These smartwatches utilize Wi-Fi and Bluetooth standards for connectivity purposes. The data collected from the smartwatches are stored in the cloud, and real-time analytics can be performed to identify customers having healthy lifestyles, while various challenges related to data privacy arise since wearables are essentially processing person-identifiable biometric information [126]. In particular, the data processing should follow regional-specific regulations, e.g., General Data Protection Regulation (GDPR) in EU [127].

Furthermore, wearable devices can be equipped with both short-range and long-range connectivity chipsets. It can be justified by the device manufacturers’ aim to enable standalone and hands-free operations for wearable devices and end-users, respectively [128]. The main long-range connectivity solutions considered for industrial wearable applications are based on low-power wide-area (LPWA) standards. As their name suggests, LPWA technologies are optimized for low power operation, thus, the long battery life in low-end wearable applications. In [120], the authors demonstrated a smart shoe that harvests mechanical and solar energy to provide it to the LoRa radio component. These smart shoes can be utilized in tracking and physical activity monitoring applications. Similar to LoRa technology, Sigfox is also among the LPWA solutions that are being used in wearable tracking systems [129].

Tracking applications are not the only services that can be provided by wearable solutions using long-range communication technologies. For instance, the AlertGPS wearable devices offer a multitude of functionalities for worker safety, including mass notifications in cases of fire, bad weather, or other emergency situations [123]. They also offer the feature of emergency calls where the worker can initiate a call with a safety agent using conventional Long-Term Evolution (LTE) cellular technology [130]. Although the adoption of licensed cellular technologies in wearable solutions has not received enough attention in the literature, the cellular IoT standards that were ratified by the 3rd generation partnership project (3GPP) to support the LPWA operations can enable wearable applications with better coverage, scalability, interoperability, quality of service (QoS), and security [19]. These cellular IoT standards include narrowband IoT (NB-IoT) and LTE-machine type communications (LTE-M). Despite being introduced in 3GPP Release 13 as part of LTE standards, NB-IoT and LTE-M fulfill the international mobile telecommunications-2020 (IMT-2020) requirements for massive machine-type communications (mMTC) and can be, as confirmed in the 3GPP study on self-evaluation towards IMT-2020 submission, certified as 5G technologies [131].

**Table 5:** Communication technologies utilized in industrial wearable applications.

Category	Technology	Frequency	Max. Data Rate	Refs.
Short-range (<100 m)	RFID	0.86 GHz	40 kbps	[132]
	Zigbee	868 MHz, 915 MHz, 2.4 GHz	20 kbps, 40 kbps, 250 kbps	[125]
	Bluetooth	2.4 GHz	1–3 Mbps	[123,133]
	BLE	2.4 GHz	125 kbps–2 Mbps	[123,133]
Mid-range (100 m–5 km)	IEEE 802.11b	2.4 GHz	1–11 Mbps	[123,134]
	IEEE 802.11g	2.4 GHz	6–54 Mbps	[123,135]
	IEEE 802.11n	2.4/5 GHz	Up to 600 Mbps	[123,135]
	IEEE 802.11ac	5 GHz	Up to 1 Gbps	[123,135]
Long-range (>5 km)	LoRa	915–928/863–870/433 MHz	50 kpbs	[136,137]
	Sigfox	868/902 MHz	100 bps	[129]
	LTE	3GPP frequency bands	100 Mbps	[130]
	LTE-M	3GPP frequency bands	1 Mbps	[138]
	NB-IoT	3GPP frequency bands	250 kbps	[139,140]

On top of the currently utilized short-range, mid-range, and long-range technologies provided in Table 5 and with the increasing attention addressed to industrial wearables, other candidate communication technologies are lately being taken into account and studied to support the novel requirements. Among these, the IEEE 802.11ah standard, also known as Wi-Fi HaLow, is considered to be an enabler of the low-power connectivity required in wearable applications [141]. Its extended range can provide wearable devices with seamless connections in challenging environments like industrial setups and, being backward compatible, is expected to allow seamless integration with higher energy efficiency [142]. Further, and on top of the cellular IoT standards for wearable mMTC, certain industrial wearable applications can have requirements [143] that are similar to the other two 5G service classes, namely enhanced mobile broadband (eMBB) and ultra-reliable and low latency communications (URLLC) [144]. For instance, AR and VR-based applications require high data rates, high reliability, and low latency and can utilize the millimeter wave (mmWave) 5G technology [145]. Other potential mmWave and terahertz technologies for industrial high-end wearable applications include IEEE 802.11ad (also called WiGig) and Visible Light Communications (VLC), respectively.

### 3.3. Localization Techniques

As mentioned, identifying the exact location of the objects is one of the most important functions performed by wearable devices. There are two types of objects in industries: machinery/equipment and personnel. Accurate positioning is key to preventing worker collision with moving machinery, exclusion of an opportunity of unauthorized access to hazardous work areas and equipment, successful evacuation, and efficient distribution of labor. However, positioning continues to be one of the most challenging problems for industrial wearable devices due to the nature of the workplace (e.g., underground, underwater) and, at the same time, high accuracy requirements. Moreover, employee location tracking also raises data ownership, security, and privacy questions, which will be explored in more detail in the next section.

All location tracking techniques could be divided into two groups: methods depending on range and range-free techniques [146]. The first group considers the conversion of various parameters to the range. It comprises time-based measurements (Time of Arrival (ToA), Time Difference of Arrival (TDoA)), angle-based measurements (Angle of Arrival (AoA), Angle of Departure (AoD), power-based measurements (Received Signal Strength Indicator (RSSI), in connection with which path loss models are used).

In the second group’s schemes, for example, in the Distance Vector-Hop algorithm (DV-HOP), anchors broadcasting their location to the whole network, and unknown nodes estimate their location based on the proximity to these known anchors (hop size and hope count). Such algorithms can be used without any additional equipment [146]. The choice between these two groups is based on the trade-off between price and accuracy: range-based techniques provide high precision, but their application is quite expensive. Range-free techniques are usually considered a cheaper and less precise alternative to the first group.

To choose the localization solution for a particular case, one should review such parameters as the environment (outdoor/indoor), coverage, power consumption, scalability, price, and accuracy.

The first question is what environment is more typical for industrial cases: outdoor or indoor. This question is essential since the different conditions dictate different approaches for these two cases [146]. While indoors is the scatter-rich environment where the path loss model’s prediction is a big challenge, outdoors usually have Line-of-Sight. For the same reason, achieving high accuracy in the first case is much more complicated than in the second. However, it is more desirable, especially during evacuations from the rubble or other emergency cases. In outdoor cases, random existing static anchor stations are usually used to determine the location, whereas in indoor cases, anchors’ deployment could require complex preliminary calculations. We further consider the techniques from both indoor and outdoor cases, see Table 6.

Usually, we want to identify the worker’s position in a relatively limited area, referring mainly to indoor localization. It is worth noticing that in such manufactures as construction or logistics, for example, just indoor localization is not sufficient. Thus, the perfect variant for some industries would require seamless connectivity, which continuously monitors the location of people/equipment/assets. However, seamless localization is still a problem: there is no localization solution for both outdoors and indoor cases and cellular-based solutions have lousy accuracy. This situation is expected to be changed with the coming 5G that, as was announced, will ensure sub-meter accuracy. However, the question is still open.

As mentioned before, localization accuracy is still a big issue, especially in the indoor environment. To improve it, engineers explore and apply different combinations of technologies as it was done in QUUPPA Intelligent Locating System where RSSI was combined with AoA Direction Finding signal processing methodology [164]. However, the declared high accuracy of less than 10 cm is offset by high cost, small coverage, and relative deployment complexity [165].

Another big issue in this area is providing an accurate localization in underground work sites. For these purposes, ground-based pseudolites (pseudo-satellite transmitters) can provide localization in industrial environments where the GPS has poor or no coverage, such as deep, open-pit mining, high water dams; urban canyons; large indoor industrial halls. Two well-known positioning solutions are LocataNet, developed by Locata Corporation/Leica, and Terralite XPS, developed by Trimble. Both use a network consisting of fixed pseudolites installed on the ground, around the perimeter of the objective; mobile receivers installed on moving equipment such as heavy engineering vehicles and aircraft [166,167,168]. These pseduolite systems operate in industrial environments such as Boddington Gold Mine (Australia), Morenci Copper Mine (Arizona, USA), White Sands Missile Range (New Mexico, USA). The main advantages are centimeter-level accuracy, coverage radius of several tens of km, obtained with just 10 pseudolites [168]. These systems require an initial complex ground deployment and set-up by the manufacturer on-site. The puseudolites need access to a power source and a clear line of sight for best precision. This solution was still not applied in the IIoT area to the best of the authors’ knowledge but should be considered a promising one. Other positioning methods proposed for underground workspaces are: ZigBee, Visible Light Communication [169], WiFi, BLE, Inertial Measurement Units [170], image-assisted person localization [171]. Most of them require a large density of beacons and stations, offering meter accuracy. For example, in the USA, since 2006, all mine operators must adopt electronic tracking systems, RFID being the most popular solution [172].

To conclude, choosing the appropriate localization techniques in each case compromises accuracy, coverage, power consumption, scalability, and price. When discussing localization techniques for industrial wearables, we need to consider that we usually deal with low throughput, low power, small size, and specific locations (underground, underwater). The position is an essential parameter for work safety providing and remains one of the main accuracy-related issues (especially in indoor and underground conditions) and smoothness of tracking.

## 4. Challenges and Future Perspective

Nowadays, the innovations’ speed has significantly increased compared with the last decades: the new technologies boost the development of another technology, facilitate our lives, and create solutions in different areas, including the enterprises’ sector. Along with the challenge of improving work efficiency, IIoT, and wearable devices, as part of it, help the industry cope with the challenge of ensuring a high safety level at work. However, the new technologies open new opportunities and bring new questions and the old challenges for which no reliable solution has been found. In this section, we summarized the most significant ones in Table 7.

In [18], the authors highlight the main challenge groups compliant with the adoption of wearable devices: technological, social, economic, data-related, and standards-related. Using the same terms, let us highlight the main challenges and areas for further investigation in industrial devices, directly or indirectly contributing to preserving human health and life.

In the framework of technological issues, several previous works pointed out the problems of finding the trade-off between functionality, battery life of a wearable device, its size and convenience for the user, adjusting one device to several users in big industries, management, and processing of the considerable amount of data produced by the heterogeneous devices [18,92]. In addition to it, it is also worth noting the problem of ensuring high accuracy of positioning, especially in indoor scatter-rich environments. Currently, one of the most spread solutions is Wi-Fi fingerprinting. However, it is quite power-consuming for industrial wearable devices. While the indoor environment is more typical for industrial scenarios, the outdoor environment is also not rare: such sectors as agriculture, logistics, forestry, etc., involve large work sites.

Moreover, some industries, e.g., construction, include both types of environment. It raises the question of the possibility of ensuring seamless localization since it is more convenient for an enterprise to use one technology in terms of price and compatibility. However, nowadays, we still do not have a reliable solution for this purpose, and cellular technology does not meet high precision requirements. Currently, active attempts are being made to close this research gap, and the 5th generation of mobile communications promises to improve the situation on this issue significantly.

Another sharp technological problem is the development of connectivity solutions and propagation models in underground work sites, especially in the sector of coal mining, which is considered as one of the riskiest places of work due to the high probability of roof-falling, the concentration of toxic gases, explosions and so on. In this case, technology should cope with low-power signals, electrical interference, multiple reflections across the corners, and at greater depth [177]. The problem of the existing solutions like Wi-Fi, Zigbee, Bluetooth, cellular technologies lies in short communication distance and high delay [178]. Simultaneously, the need to develop a reliable technology for this field is undeniable and creates a direction for further research.

Talking about large worksites and the deployment of wearable sensor networks supporting many environmental and wearable devices, we will still meet the issues of power consumption and supply due to the necessity to track and sustain a charge of a large number of devices. Substantial efforts over the past decades resulted in the development of long-lived batteries, which partially resolved the challenge. However, in some industries, the power consumption level is still very high compared to energy provision. One of the promising directions in this field is the energy harvesting approaches of light, rainwater, motion, and temperature gradients. In work [136], the authors designed a micro-power manager that can obtain energy both indoors and outdoors due to solar panels embedded in the construction of the device and thus constantly support sensor node feeding. Similarly, nowadays market offers wearable devices that can extract energy from the sun, for example, T-shirts [194] or smartwatches [195], however, for example, for the mining industry, it is not an option.

The next problem is related to privacy and security. It covers three groups simultaneously: technological, data-related, and social. Confidentiality of the data is a big question in this area: wearable devices become subject to a wide range of external attacks due to communication with another device, limited bandwidth, and processing power [180,182]. Various approaches are applied to improve the technological part of security and privacy challenges, and one of the most actively studied is ECC, the main advantage of which from the point of view of wearable devices is light weight [180,181]. However, the current world situation shows that existing solutions are not sufficient to ensure security in industries. For example, mining companies are of great interest for espionage, as they contain data on the location of valuable minerals. According to [196], 54 percent of companies in the mining/metal industry were attacked in 2019, and these numbers tend to grow. This state of affairs sets another relevant direction of development in the field of industrial wearable devices.

The question of location data privacy, namely, finding the trade-off between the accuracy of positioning of the worker and confidentiality, can be singled out separately. The exact location is essential in industries, especially during evacuation sessions. However, as was mentioned before, workers feel nervous being tracked all the time, consequently, the likelihood of error increases. Significantly, the localization could be executed either on the device itself or in the centralized processing point [185]. The second option requires additional obfuscation to provide the desired level of privacy for the user. The authors of [183] discuss the various obfuscation techniques to overcome this issue.

Data-related and social groups consider security and privacy issues from the side of data ownership questions [18,197]: how the data is distributed, who has access to it, and how it could be used. Lack of information about the flows of data collected in the workplace builds mistrust of the technology. M. Kritzler et al. in [190] state that this problem could be eliminated by a clear explanation of the purposes of the implementation of wearable technology in the enterprise. Since most employees do not share their personal information with the employer, this method is not effective, and this problem also remains on the list of research agendas for next years.

The last main challenge in industrial wearables that we would like to address here is social resistance to adopting technology. For any innovation in modern society, the level of acceptability is even more important than the corresponding benefits and usefulness [198]. Workers feel constant pressure being monitored all the time, which increases their stress level and, consequently, the probability of injury. Also, they afraid that the collected by wearables raw information, which they cannot control, improve or modify, may become a reason for dismissal or fines. Some workers cannot quickly learn how to use the wearable device and do not want to spend additional time on it, considering the traditional way of the work process as the most convenient and the only possible. The literature and practice offer several options to improve the situation, such as more friendly interfaces, video guidance, constant support. V. Jacobs et al. in [189] distinguish the row of factors that predicts the level of acceptance of industrial wearables and suggests a list of actions that should accelerate the process.

In summary, the above discussion identifies some research directions that should be explored during the next decades to accelerate the adoption of wearable technology in industries and, thus, increase work safety. Table 7 gathers the key challenges in the field of industrial wearables and general issues which are specific to the area of wearable technology.

## 5. Conclusions

Modern wearable devices already offer several opportunities to maintain occupational safety in the work environment rife with various hazard sources. This work identifies four key functions (monitoring, supporting, training, and tracking) and ten sub-functions, showing which wearables can directly or indirectly contribute to preserving workplace safety to assess wearables’ use cases and benefits for industrial work safety. Additionally, this article summarizes and classifies metrics collected by wearable devices (12 metrics related to the body and 8 metrics related to the environment), thereby showing how enterprises can control workplace safety from both personnel and environment perspectives.

It is essential to highlight that discussed modern techniques for communication, localization, and privacy protection in wearable technology still cannot fully cover all industries’ needs. The level of social resistance is still considered very high, slowing down the widespread adoption of wearable devices in enterprises. The present article summarizes the key open challenges (technological, data-related, standard-related, economic, and social) and suggests possible solutions, pointing to a wide field for future research on industrial wearable solutions for occupational safety.

## Figures and Tables

**Table 1:** Classification of industrial wearable functions.

Function	Sub-Functions & Description
**Monitoring (M)** Fitness trackers, smart rings, smart glasses, patches/ sensors attached to the body, smart clothing, implantable wearables, etc.	**Monitoring and control of vital parameters of workers.** Information about vital parameters (heart rate, blood pressure, body temperature, brain activity, etc.) gives the employer (organization, safety manager, administration) and the worker himself an idea on the readiness of the latter for the work process from a physical, less often psychological, point of view [31,32]. **Monitoring of environmental parameters at workplaces.** Knowing such parameters as the temperature at the worksite, atmospheric pressure, level of radiation, and so on allows an organization to control the overall environmental situation at the factory, prevent emergencies, timely organize the evacuation of people, provide a worker with proper Personal Protective Equipment (PPE). Moreover, by combining environmental and vital parameters, it is possible to track environmental impacts on human health in hazardous industries such as the chemical industry [33,34].
**Supporting (S)** Exoskeletons, patches (to control the position of the body when lifting heavy objects), wearable robots	**Increasing the physical capabilities of the workers.** Some industries envisage lifting and transferring heavy objects, which is often associated with musculoskeletal injuries. Wearable items such as exoskeletons support the musculoskeletal system to prevent damage [35,36,37]. **Facilitating communication between workers.** Due to their small size, weight, and comfortable attachment to the wearer’s body, wearables such as headsets with embedded hand-free microphones, for example, are much more convenient than phones and able to provide communication between workers without distracting them from the work process. (hand-free microphones embedded in headsets/helmets) [38]. **Simplification of information management.** Wearable devices provide secure transmitting, storage, displaying information, and fast access to documents and notifications [39]. **Performing industrial design.** The use of AR enables creating virtual diagrams and graphs that facilitate better understanding by workers [40].
**Training (Tn)** Smart glasses, helmets, heads-up display	**Training of the workers.** Some wearable devices can track the correctness of the actions performed by the worker, providing him with a detailed report (for example, determining the correct posture using biomechanical analysis). The worker can analyze his mistakes to prevent them in the future. Moreover, using Virtual Reality (VR) and Augmented Reality (AR) helmets, it is possible to train workers on complicated operations before performing them in reality, thereby reducing the likelihood of injury [36,40].
**Tracking (Tc)** Smart bracelets, smart clothes, smart boots, digital pedometer, etc.	**Monitoring of location parameters of workers.** The worker’s location is one of the most important parameters when we are talking about ensuring work safety in the industry. By knowing each employee’s location, the safety manager can efficiently organize evacuations, distribute help and workforce, prevent unauthorized access to the worksite or equipment, and so on [41]. **Preventing struck by moving machinery.** Tracking of object locations and proximity detection sensors allow avoiding a collision that is one of the most spread accidents in industries [33,41]. **Creating a comprehensive picture of the whole production process.** Thanks to wearables, managers can see the real-time location of workers and equipment, which allows them easily redistribute labor between operating sites and more effectively allocate resources [41].

**Table 2:** Industry branches and inherent industrial wearable functions.

Industry Branch	Functions (See Table 1)	Examples of Applications
Mining	M, Tc	US-based Guardhat to prevent injuries combining rugged helmets with microphones, cameras, and track sensors [42]. Guardhat is currently integrated into a lot of mining operations.
Chemical	M, Tc	MyExposome developed wristbands that can detect chemical exposures during the day [34].
Forest products, construction	M, Tc, S	SolePower company released smart boots [41]. It is equipped with many various modules, particularly GPS and RFID, to determine the wearer’s location. Besides, they are very durable, so they can easily withstand many ordinary adverse external influences.
Crude oil production	M	Smart Helmet by VRMedia Srl. was applied by the third-largest oil and gas service company in the world Baker Hughes to reduce downtime and increase safety at the workplace [43].
Transportation, Shipping	M, Tc, S	Kinetic presents a wearable device called REFLEX that is equipped with sensors and modules performing biomechanical analysis [36]. It is worn on the belt or waistband and can determine whether the posture is correct or not and notify the user by vibration when risky movements arise.

**Table 3:** Human body-related metrics.

Metric	Description	Example Accuracy	Examples
Blood pressure	The pressure that blood puts on the walls of blood vessels. There are systolic or upper (normal value: less than 120 mmHg) and diastolic or lower (normal value: less than 80 mmHg) blood pressure [45]. The average working range of blood pressure sensors is 0–320 mmHg [46,47].	86% [48]	Arm cuffs with attached sensors [49], cuff-less blood pressure sensors [50]
Calorie	A unit equal to the amount of heat needed to increase one gram of water temperature by one degree Celsius. There are several ways how to calculate the number of calories (e.g., based on the number of steps or heart rate [51]), resulting in a wide range of wearables providing this function.	>91% (walking); >90% (running) [52]	Accelerometers, pressure sensors in fitness bracelets, smart shoes [51], etc.
Electro- cardiogram (ECG)	The electrical activity of the heart [49]. The unit of measurement is Volts. ECG is the main diagnostic method for detecting cardiovascular diseases such as hypertrophy, heart attack, arrhythmia [53].	>90% [54]	Skin electrodes [49] in clothes [55], chest straps [56], etc.
Electro- encephalo- gram (EEG)	The electrical activity of the brain [57]. The unit of measurement is Volts. EEG is used to identify and predict brain-related diseases (e.g., Alzheimer’s disease, epilepsy, dementia) [58]. In addition, it also used for the emotion detection [59,60]. Until now, ECG, EEG, and EMG are performed mainly in medical institutions. However, there are already some wearable devices on the market for such measurements.	>86% [59,60]	Headset [57,61]
Electro- myography (EMG)	The electrical activity of the muscles [49]. The unit of measurement is Volts. When measuring EMG, the critical point is the exact position of the electrodes on the muscles. EMG is used to identify the muscle traumas and monitor the recovery tendency after such traumas [62].	>90% [63]	Skin electrodes [49] embedded in bracelets, waist straps [64], clothes [65].
Glucose	The level of sugar in the blood. It is measured in grams per liter or moles per liter. High glucose level identifies diabetes, the symptoms of which are quite wide, ranging from visual impairment to increased fatigue and depressive episodes [66,67]	>95% [68]	Strip-base [69], implantable [70] glucose sensors, smart tattoos [71]
Heart rate and pulse	Heart rate is the number of heartbeats per minute. Pulse is the number of vibrations of the aortic walls. Pulse may be a less accurate characteristic in pathologies (for example, extrasystole) since not all heartbeats lead to the formation of a pulse wave [72]. Critical boundaries usually range between 40-200 beats per minute and depend on current activity, gender, age, health, type of activity, etc.	>76% [73]	Pulse oximeter [74], chest [75] and wrist straps [69], fitness bracelets
Heart sounds	Sounds that appear due to a change in blood flow, vibration of the surrounding tissues of the heart, and large vessels. The conventional way to measure heart sounds is phonocardiograph [76]. However, there are already some wearable solution [77].	>80% [77]	Wrist band [77]
Location- related metrics	Metrics related to identifying the object’s position: coverage, accuracy, power consumption, price of the wireless technology, etc. Localization technologies are considered in more detail in Section 3.3.	NA	Wide range of wearables
Motion- related metrics	This metric refers to identifying the parameters of the human movements that are also called biomechanical analysis [78,79]. The range of the wearables for which this metric is used is very wide since the measured parameters could be very different: from detection of the velocity and speed to determining if the posture correct or not.	NA	Accelerometer, gyroscope [80], exoskeletons, pressure insoles, e-textile [78]
Perspiration or sweat	A liquid excreted from the skin’s sweat glands [81]. Sweat is the second body fluid after the blood that contains the richest range of biomarkers like glucose, pH, cortisol, etc. [82]. Usually, this metric is used in sport or healthcare areas.	>99% [83]	Sweat collectors, skin patches [82], smart watches [84]
Temperature	A measure of the ability of the body to generate heat [49]. However, the normal temperature range for a healthy human is 36.16–37.02 °C [85], and the widest recorded range is 24–44 °C [86], usually the range of the wearables measuring temperature is wider.	>99% [87]	Temperature sensors [88,89] and skin patches [90]

NA—Accuracy is not specified for metric groups.

**Table 4:** Environment-related metrics.

Metric	Description	Examples
Air Quality Index (AQI)	An index shows the degree of air pollution in a certain area [91]. It is calculated based on measured concentrations of pollutants and government-set limits for those concentrations. The list of measured pollutants can include ozone, carbon monoxide, sulfur dioxide, nitrogen dioxide, dust, etc. The possible values of the index are between 0 to 500. The scale is divided into ranges, usually 5 or 6, each corresponding to a specific air quality rating, from good to hazardous. The influence of high AQI levels (101 and above) on the human body varies depending on the predisposition (great age, heart/lung diseases), and could lead to such diseases as lung cancer, stroke, pneumonia, etc. [91]	Gas sensors (e.g. CO2 sensor [92])
Atmospheric pressure	The pressure exerted by the weight of the atmosphere on the surface (of the Earth or another planet) below it [93]. This metric is necessary for jobs in low (pilots) or high (divers) barometric pressure conditions. On average, the measurement range of pressure sensors is from 300 to 1100 hPa with an error of 0.5 hPa. Extra low or extra high atmospheric pressure cause respiratory, heart, neurological changes, barotraumas, decompression illness, etc. [94,95]	BMPxxx sensor group [92], barometers embedded in bands, smartwatches, glasses [96], etc.
Light intensity	The strength of light produced by a specific lamp source measured in lux [97]. The light intensity’s recommended levels in different cases can be found in the document issued by the National Optical Astronomic Observatory [98]. Both excessive and insufficient lighting in the workplace can lead to visual impairment. A wide range of health effects of lighting is observed in working conditions at night or in underground sites, including various types of cancer, irregular sleeping habits, and cardiovascular disorders [99,100]. Significantly, an insufficient illumination intensity is considered as causing an additional increase in the rate of accidents in low-light environments, such as construction areas, warehouses, and tunnels [101].	Motion, traffic, ambient light sensors [20], e.g. [102]
Noise level	The amplitude level of the undesired background sound [103] is measured in dBA. Constant sound above 80 dBA leads to the physiological effects and above 100 dbA—to the hearing damage, [104].	Sound sensors, dynamic microphones [20]
Radiation	An energy from a nuclear reaction [105]. Nowadays, it is measured in Sv (Sievert). However, rem (roentgen equivalent man) units also could be found in the literature. US Nuclear Regulatory Commission has set a radiation limit of 5 rem or 0.05 Sv [106]. Even an acceptable level of radiation during a long period of time (what is typical for radiation industry employees) can be a reason for irreversible changes in the body, in particular, the risk of cancer increases. High doses lead to the vomit, skin burns, can cause death [107]	Radiation detectors [108]
Relative Humidity	The amount of water that is present in the air compared to the greatest amount it would be possible for the air to hold at that temperature [109]. The hygienic norm of relative humidity for humans is 30–60%. With low humidity, the body becomes dehydrated, and the risk of bacteria entering the human organism increases. High humidity can cause overheating, increased perspiration rate, and promotes the appearance of allergens (mold, fungi, dust mites) [110,111].	Temperature/humidity sensors [112]
Temperature	Ambient temperature, which is most often expressed in degrees Celsius. The the typical range of temperature sensors is −40 to 125 degrees Celsius °C [82]. The survival limit for the person is between −40 °C [113] and 48 °C. For the best performance the optimal ambient temperature is 22–26 °C [114].,	Temperature/humidity sensors [82,112]
Ultraviolet index (UVI)	An index shows the degree of ultraviolet radiation from the sun at a particular time and place. For measuring UVI World Health Organisation (WHO) proposed a linear scale beginning from 0 and without an upper border. There are 5 ranges: low (UV: 1–2), moderate (3–5), high (6–7), very high (8–10), extreme (11+) [115]. The sun exposure with UV higher than 7 can lead to serious damage of eyes (e.g., snowblindness), skin (burns, skin cancer, skin aging), and overall immune system [116].	UV radiometers and dosimeters embedded in wrist bands, smartwatches, clips, etc. [117]

**Table 6:** Comparison of localization solutions.

Technology References	Localization Techniques	Typical env-t.	Accuracy	Additional Details
GNSS [147]	Time-based	Outdoor	cm-level	Global, not applicable indoors, still considered as high consuming for industrial wearabes, however, some companies already started to present ultra-low power GNSS [148]
Wi-Fi [149,150]	Time-based, Angle-based, Power-based	Indoor	m-level	Nowadays, Wi-Fi fingerprinting (FP) is one of the most promising localization approaches for industrial wearables due to good accuracy and relatively low cost. The disadvantage of this approach is high consumption in terms of power and efforts spending on the training step
BLE [151]	Time-based, Power-based	Indoor	m-level	Another perspective technology for indoor localization in IIoT [152] with such advantages as easy deployment, low cost, and low power consumption. However, the accuracy of the method is not very high, and supplementary algorithms are required to improve it [153].
UWB [150,154]	Time-based, Angle-based	Indoor	cm-level	This method provides the highest accuracy of the localization, requires not much power, has immunity to fading, applicable even in the case of underground worksites. However, it is hard to deploy [155,156].
LoRa [157,158]	Time-based, Power-based	Outdoor	100 m-level	The key advantages of this technology are coverage range (up to 20 km), low consumption, bigger stability than in case of WiFi and BLE [159]. However, low accuracy prevents the spread of the technology as a localization solution.
Sigfox [160,161]	Power-based	Outdoor	100 m-level	Advantages and disadvantages are similar to LoRaWAN technology.
RFID [132,162,163]	Power-based	Indoor	cm-level	This technology ensures high precision but in a low range (approx. 15 m).
	Angle-based	Indoor	m-level	Less accurate than passive RFID but has bigger range (approx. 150 m).

**Table 7:** Key challenges in the adoption of industrial wearables.

Challenge	Groups	Refs.	Possible solutions
Localization accuracy indoors/outdoors	T	[173,174]	Applying of ML algorithms to identify missing values, predict the number of obstacles and distance between RX and TX
		[175,176]	Seamless localization to provide smooth tracking both indoor and outdoor
Connectivity solutions and propagation models for underground work sites	T	[177,178]	Application requirement-based selection of connectivity solutions, developing of empirical and industrial environment-specific propagation models with on-body/off-body/body-to-body communications
Power consumption and supply of a big amount of devices	T	[136,179]	Energy harvesting approaches: harvesting from the sunlight, motions, temperature gradients, etc.
Privacy and security	T, S, D	[180,181]	Elliptic Curve Cryptography (ECC) and other lightweight cryptography
		[182]	Development of strong authentication schemes
Location data privacy	S, D	[183,184]	Adding noise to the exact coordinates on the device side before transmitting it to the cloud
		[185,186]	Transmission of the location-related function instead of the coordinates
Social resistance	S	[18,92]	Development of the simple and detailed manual, video guidance and provision of the constant support to eliminate the problem of low technical skills of users;
		[187,188]	Usage of Technology Acceptance Models (TAM) to estimate key factors affecting the level of social resistance and rearrange the process implementation of the technology accordingly
		[189]	Involving of the employees in the process of the choice of wearables
		[190]	Data flows transparency
Heterogeneity of the IIoT devices	D, SD	[191]	Application of the data fusion approaches on the hardware level, seamless integration on the protocol level
Placement of preprocessing and processing entities	D	[192,193]	Optimization of data placement, dynamic computation resource allocation, computation offloading techniques.
High cost of wearables and its coupling with other technology in big industries	E	[18]	Development of one-size wearable to be used for data acquisition by different workers during different shifts

D—Data-related; E—Economic; S—Social; SD–Standard-related; T—Technological.

## Abbreviations

The following abbreviations are used in this manuscript:

AoA	Angle of Arrival
AoD	Angle of Departure
AQI	Air Quality Index
BLE	Bluetooth Low Energy
DV-HOP	Distance Vector-Hop
ECG	Electrocardiogram
EEG	Electroencephalogram
eMBB	Enhanced Mobile Broadband
EMG	Electromyography
GNSS	Global Navigation Satellite System
GPS	Global Positioning System
HAV	Hand-arm Vibration
IEEE	Institute of Electrical and Electronics Engineers
IIoT	Industrial Internet of Things
ILO	International Labor Organization
IoT	Internet of Things
IWSN	Industrial Wireless Sensor Networks
LoRaWAN	Long-Range Wide-area Network
LPWAN	Low-power Wide-area Network
MDPI	Multidisciplinary Digital Publishing Institute
mMTC	Massive Machine-type Communications
NB-IoT	Narrowband IoT
RFID	Radio Frequency Identification
RSSI	Received Signal Strength Indicator
TDoA	Time Difference of Arrival
ToA	Time of Arrival
UE	User Equipment
URLLC	Ultra-Reliable and Low Latency Communications
UVI	Ultraviolet Index
VLC	Visible Light Communications
WHO	World Health Organization
